# Tetraplegia After Aortic Arch Surgery in Patients With Cervical Spinal Stenosis

**DOI:** 10.1016/j.atssr.2022.09.005

**Published:** 2022-09-20

**Authors:** Ryuki Yamada, Takuto Maruyama, Shuntaro Ito, Hiroaki Yamamoto, Shinichiro Abe, Yoshinori Enomoto, Soichi Asano

**Affiliations:** 1 Department of Cardiovascular Surgery, Chiba Cerebral and Cardiovascular Center, Chiba, Japan; 2 Department of Cardiovascular Surgery, Kimitsu Central Hospital, Chiba, Japan

## Abstract

We report 2 rare cases of tetraplegia after total aortic arch replacement with frozen elephant trunk that were attributable to cervical spinal stenosis. Cervical spinal stenosis can increase the vulnerability of the spinal cord to ischemia and may increase the risk of spinal cord injury in operation with circulatory arrest.

Spinal cord injury (SCI) after aortic operation is a serious complication, but most reported cases involve paraplegia. We report 2 rare cases of tetraplegia after total aortic arch replacement with frozen elephant trunk (FET) that may have been caused by cervical spinal stenosis.

## Case Reports

### Patient 1

The patient was a 79-year-old man. Computed tomography (CT) showed a large aneurysm (50 mm) in the aortic arch. He had a history of hypertension. He had occasional numbness of the fingertips of his left hand, but this was not apparent before operation.

The patient was intubated without any problem. His head was kept in an appropriate position to prevent hyperextension of the neck. Cardiopulmonary bypass was established from the right atrium to the ascending aorta. After the induction of circulatory arrest (body core temperature <25 °C), a balloon-tipped cannula was inserted into 3 aortic arch branches from inside the aorta, and antegrade cerebral perfusion was started. The balloon catheter line pressure was monitored, and cerebral oxygen saturation was monitored bilaterally from the forehead using near-infrared spectroscopy. The aorta was transected transversely, and a 25 × 120-mm Frozenix (Japan Lifeline) was deployed. Open distal anastomosis was achieved with a 28-mm 4-branched J graft (Japan Lifeline). Lower body reperfusion was reinitiated after 62 minutes of circulatory arrest, and the left subclavian artery was reconstructed to the graft branch. Proximal anastomosis was performed, then the remaining cerebral branch was reconstructed individually. The operation finished uneventfully after 316 minutes.

**Figure 1:**
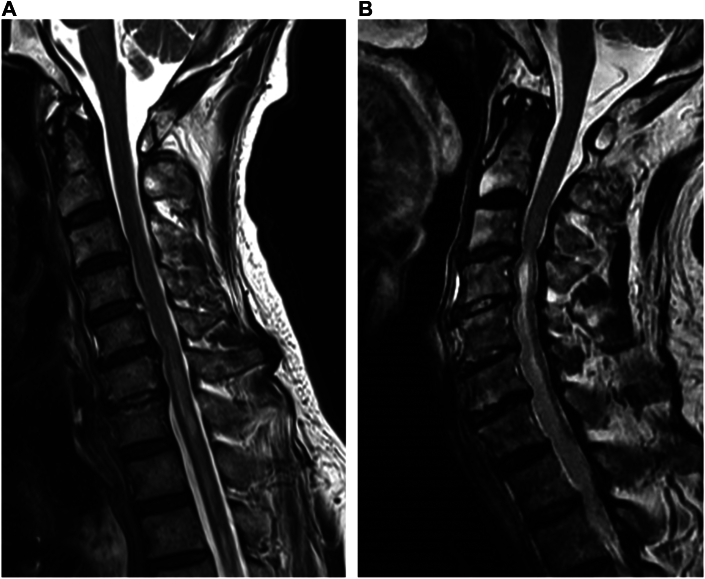
Sagittal T2-weighted magnetic resonance images show spinal cord compression at (A) C5/6 and (B) C4/5 and C5/6 and an increased signal intensity of the spinal cord at (A) C6-T2 and (B) C5-T2.

**Figure 2:**
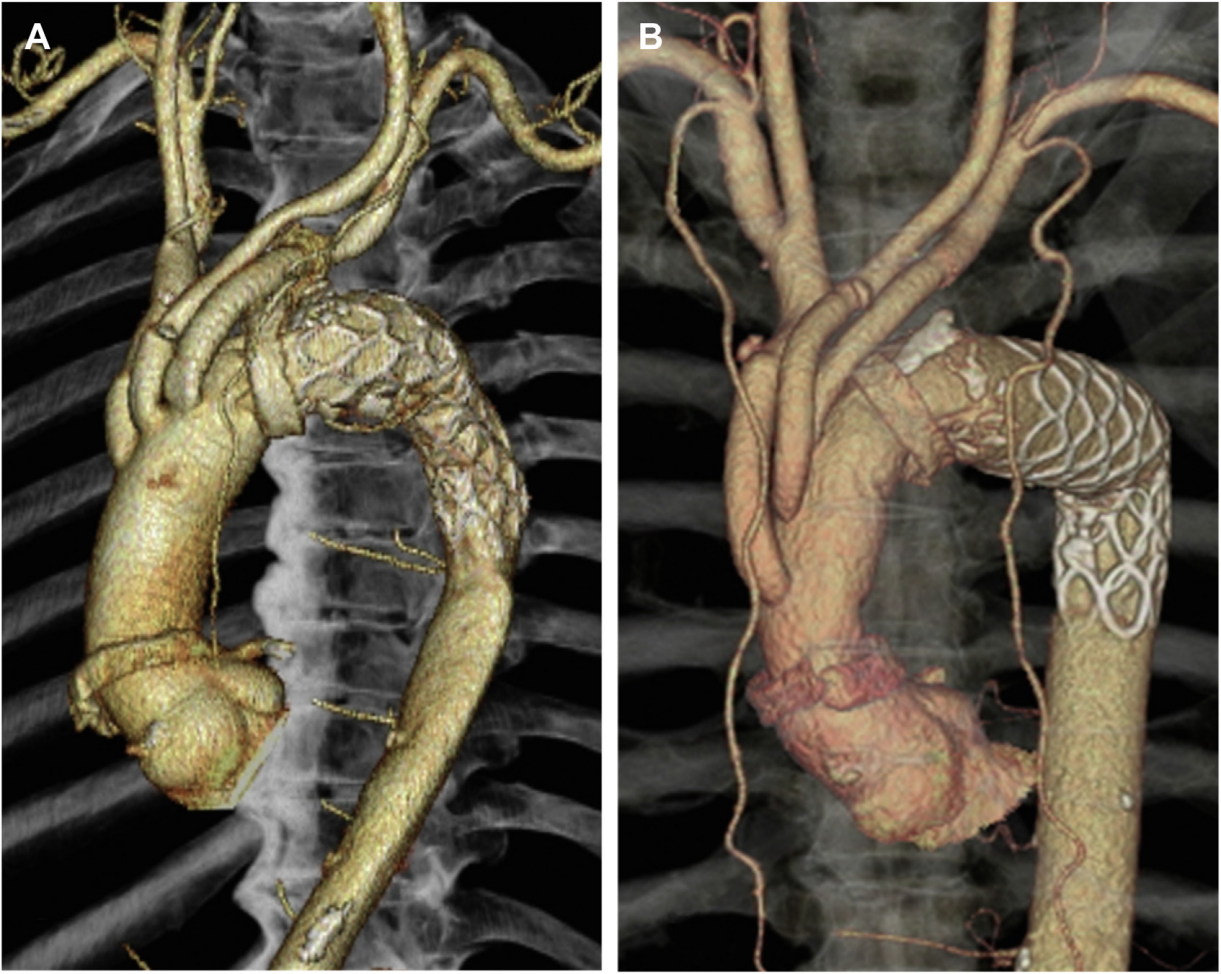
Postoperative 3-dimensional computed tomography images. (A) Patient 1 and (B) patient 2.

After return to the intensive care unit, the patient’s status remained stable, and his mean blood pressure remained >75 mm Hg. The patient awakened 3 hours later, but his extremities were flaccid. We tentatively diagnosed SCI. The patient underwent cerebrospinal fluid drainage for 3 days and received blood transfusion, norepinephrine, methylprednisolone, and naloxone. Extubation was performed the day after surgery. On postoperative day (POD) 3, he was able to move his right lower extremity slightly. On POD 9, magnetic resonance imaging (MRI) was performed. A cord compression lesion was found at C5/6, with extensive high signal intensity on T2-weighted imaging, indicating ischemic injury at C6-T2 (Figure 1A). No intracranial infarct or hemorrhage was observed. CT angiography showed the FET deployed at the T4-T6 level (Figure 2A). Rehabilitation was continued until the patient was transferred to a rehabilitation hospital on POD 59. At that time, he could walk with minimal support but could not achieve a full grip with his left hand.

### Patient 2

The patient was a 66-year-old man. CT showed a large aneurysm (59 mm) in the aortic arch. He had a history of hypertension. He had no symptoms suggestive of cervical spondylosis myelopathy.

As is routine at the hospital, the patient was intubated with a video laryngoscope, and a pillow was not inserted under his shoulder; thus, the neck was kept in an appropriate position during operation. The operative strategy was almost the same as for patient 1. A 29 × 120-mm Frozenix was used as an FET. Operation was uneventful. The duration of circulatory arrest and of the total operation was 74 and 450 minutes, respectively.

After return to the intensive care unit, the patient’s status remained stable, and his mean blood pressure remained >75 mm Hg. After extubation (9 hours postoperatively), the patient complained of complete loss of the motor function of his extremities, with sensory loss below the shoulder. The patient underwent cerebrospinal fluid drainage for 3 days and was treated as previously described. On POD 16, MRI revealed spinal cord compression at C3/4 and C4/5, with extensive high signal intensity at C4-C7 on T2-weighted imaging (Figure 1B). CT angiography showed the FET deployed at the T4-T6 level (Figure 2B). Unfortunately, he showed no improvement during his hospital stay and was transferred to a rehabilitation hospital on POD 36.

## Comment

SCI is a complication of aortic operation. Risk factors for SCI include prolonged circulatory arrest at ≥28 °C, perioperative hypotension, FET deployment more distal than T8, diabetes, and advanced atherosclerosis.^1^^,^^2^ There are few reports of tetraplegia after aortic operation. The 2 cases reported here included none of these risk factors. However, SCI occurred at the cervical level, which is considered ischemia resistant,^3^ leading to tetraplegia. We suspected a relationship between spinal cord compression associated with cervical spinal stenosis and SCI because both injuries occurred from the site of spinal cord compression.

Neurologic dysfunction of the cervical spinal cord can be due to direct compression, spinal cord ischemia, and infection. There are some reports of cervical SCI in asymptomatic or undiagnosed preoperative cervical spinal stenosis after nonaortic operation, but most cases are attributed to hyperextension of the neck.^4^^,^^5^ Keeping the neck in an appropriate position during operation is reported to be especially important because extension of the neck narrows the spinal canal and can lead to SCI. In both of these cases, the neck was kept in an appropriate position; thus, hyperextension was unlikely to have been the cause of SCI.

Regarding spinal cord perfusion, we cannot rule out the possibility that a circulatory arrest time of 62 and 74 minutes, respectively, contributed to SCI, but we do not assume that significant hypoperfusion occurred because selective cerebral perfusion was adequately performed, no abnormalities occurred during monitoring, and the circulatory arrest time was not too long. An animal study showed that the spinal cord is vulnerable to ischemia and is easily injured in the presence of compression.^6^ Thus, it is possible that the inevitable slight spinal cord hypoperfusion associated with circulatory arrest caused SCI in these cases with cervical spinal stenosis.

The evaluation of cervical spinal stenosis has been proposed on the basis of the anterior-posterior diameter of the spinal canal on MRI; anterior-posterior diameter of <9.9 mm is considered to be associated with a high risk of spinal cord compression.^7^ The diameters of the 2 cases were 8.4 mm and 6.2 mm. Thus, the patients may have been at high risk for cervical SCI.

The rate of cervical spinal stenosis increases with age. Symptoms of myelopathy depend on the degree and location of spinal cord compression, including numbness, loss of dexterity, and gait disturbance. Patient 2 was asymptomatic, whereas patient 1 had slight numbness in his left fingertips, which may have been a symptom associated with spinal cord compression. The slow recovery of his left hand, even after rehabilitation, may indicate the primary site that caused the SCI. As some reports suggest,^4^^,^^5^ neurologic symptoms should not be overlooked, and a close cervical examination is indicated before operation in cases of suspected cervical spinal stenosis.

This report suggests that patients with cervical spinal stenosis, including asymptomatic cases, are at high risk for cervical SCI in operation with circulatory arrest. Although rare, it is a devastating complication; thus, the identification of high-risk patients and conduction of preoperative screening are important.

## References

[1] Preventza O., Liao J.L., Olive J.K. (2020). Neurologic complications after the frozen elephant trunk procedure: a meta-analysis of more than 3000 patients. J Thorac Cardiovasc Surg.

[2] Katayama K., Uchida N., Katayama A. (2015). Multiple factors predict the risk of spinal cord injury after frozen elephant trunk technique for extended thoracic aortic disease. Eur J Cardiothorac Surg.

[3] Rahman M., Rahman S., Siddik A.B. (2020). A review on the pathophysiology and management of anterior spinal artery syndrome. J Spine Res Surg.

[4] Xiong W., Li F., Guan H. (2015). Tetraplegia after thyroidectomy in a patient with cervical spondylosis: a case report and literature review. Medicine (Baltimore).

[5] Li C.C., Yie J.C., Lai C.H., Hung M.H. (2013). Quadriplegia after off-pump coronary artery bypass surgery: look before you place the neck in an extended position. Cardiovasc Anesth.

[6] Gooding M.R., Wilson C.B., Hoff J.T. (1975). Experimental cervical myelopathy. Effects of ischemia and compression of the canine cervical spinal cord. J Neurosurg.

[7] Ivana K., Milos K., Zdenek K. (2016). Prevalence and imaging characteristics of nonmyelopathic and myelopathic spondylotic cervical cord compression. Spine (Phila Pa 1976).

